# Nanobubble nucleation by pulsed laser illumination of colloidal gold nanoparticles

**DOI:** 10.1038/s41598-024-81831-y

**Published:** 2024-12-16

**Authors:** Yatha Sharma, Claus-Dieter Ohl, Juan Manuel Rosselló

**Affiliations:** 1 Institute of Physics, Soft Matter Department, Otto-von-Guericke University, Universitätsplatz 2, 39106 Magdeburg, Germany; 2 Faculty of Mechanical Engineering, University of Ljubljana, Aškerčeva 6, 1000 Ljubljana, Slovenia

**Keywords:** Laser, Nanobubbles, Plasmonic bubbles, Shockwave, Nanoparticles, Nanoscience and technology, Optics and photonics, Physics

## Abstract

This study expands upon a technique our team previously developed for generating nanobubbles on demand with a collimated pulsed laser beam. This work highlights how the controlled addition of gold nanoparticles enhances nanobubble generation efficiency in water, even at laser intensities well below the threshold for multiphoton ionization. Specifically, we investigated the influence of nanoparticles of three distinct sizes on the laser fluence threshold for bubble nucleation and the lifetime of the resultant nanobubbles. Our findings confirm that nanoparticles with a diameter of 60 nm exhibit the greatest nucleation efficiency, achieving nearly 45 % at a fluence threshold of around $70\,(\pm \,11)\,\hbox {mJ/cm}^2$. Interestingly, nanoparticle size did not impact the nanobubble lifetime.

## Introduction

Stable nanobubbles would offer exciting possibilities for gas storage in liquids, facilitating enhanced gas concentrations for various applications such as surface cleaning^1^, hydroponics^2^, targeted drug delivery^3^, and bioimaging^4^. However, establishing a reliable method for producing nanobubbles and understanding the basis of their stability have been open questions until now. Recently, Rosselló and Ohl^5^ have showcased an on-demand, unambiguous generation of nanobubbles in water by illuminating the liquid with a collimated pulsed laser beam^5^. The intensity used is significantly lower than the reported threshold for multiphoton ionization^6^. Initially, the nanobubble generation mechanism was unclear. However, it was hypothesized that pollutants such as mineral or metallic particles are a likely cause of bubble nucleation. Subsequent studies by^7,8^ confirmed this hypothesis, suggesting that heating of nano-sized contaminant particles through linear absorption of laser light induces a phase transition and leads to bubble generation.

In the present work, we expand upon this hypothesis by deliberately adding nano-sized light-absorbing particles of known properties to a liquid in a controlled fashion. Gold nanoparticles (GNPs) are particularly suitable. Their strong absorption due to the plasmon resonance leads to the vaporization of a liquid shell surrounding the particle when exposed to sufficiently high laser fluence^9–12^. In the literature, this phenomenon is referred to as thermoplasmonic nucleation^13^. Pulsed laser-illuminated GNPs have been used for cancer diagnosis and treatment^14–16^ and for targeted drug delivery^17^ and are thus a well-studied subject. Yet, in biomedical applications, ensuring the biosafety of a nanoplasmonic system necessitates safeguarding the integrity of the metal nanoparticle, which acts as a thermal source for controlled bubble generation. Consequently, selecting the optimal conditions for nanobubble generation around the nanoparticle becomes crucial to achieve controlled tissue damage with minimal laser fluence, thus preserving both the nanoparticle structure and surrounding tissues.

In this study, we explore how the concentration and size of the gold nanoparticles affect the nucleation efficiency of nanobubbles when exposed to nanosecond laser pulses near their plasmon resonance wavelength. Nucleation efficiency is determined by comparing the ratio of observed bubbles to the number of particles present in that same liquid volume. This efficiency is measured as a function of the minimum fluence necessary for nanobubble nucleation for each particle size. In addition, we report the nanobubble lifetimes and size distributions for the different particle sizes.

## Experimental method

**Figure 1:**
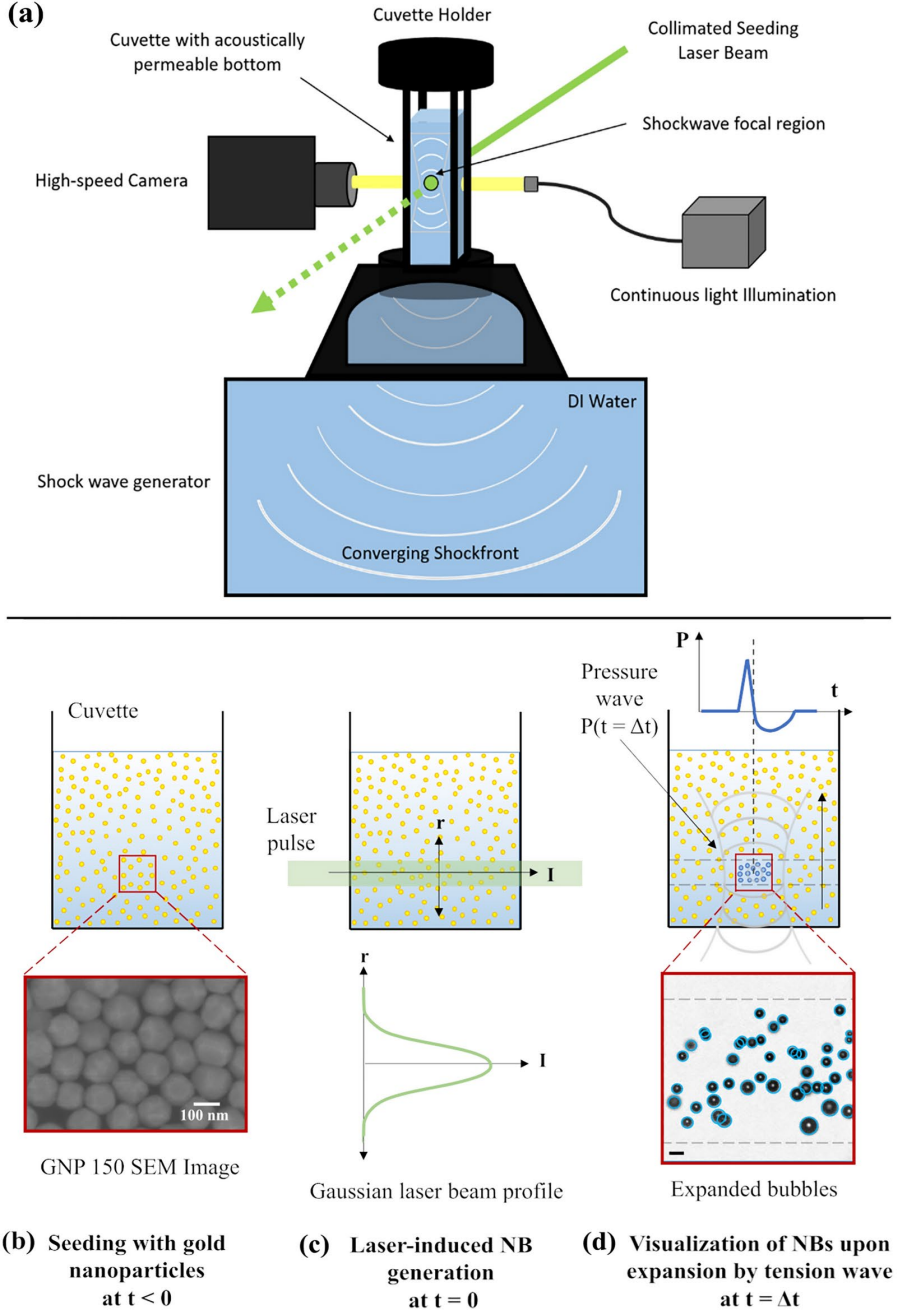
(**a**) Experimental setup. (**b**–**d**) Sequence illustrating the key steps of the experimental method. (**b**) Front view of the cuvette filled with the sample containing GNPs of 150 nm diameter. The inset shows the SEM image of the same nanoparticles. (**c**) A collimated laser pulse beam of Gaussian profile passes through the liquid sample at $t\,=\,0$ and generates nanobubbles. (**d**) At $t\,=\,\Delta$t, the seeded bubbles expand upon the arrival of the rarefaction wave produced by the shockwave generator. The pressure wave profile (top) comprises a fast-rising shock front followed by a rarefaction phase after $1.32\,(\pm \,0.05)\,{\upmu }$s. The inset (bottom) shows the expanded bubbles visualized using high-speed microscopy. The bubbles (encircled in blue) are counted using an in-house bubble detection script based on the Hough transform. Here, the frame was captured at $14.8\,{\upmu }$s after the laser seeding. The laser pulse energy in this case was 15.26 mJ and negative pressure of the rarefaction wave was − 5.5 MPa. The scale bar in the inset represents $200\,{\upmu }$m.

The experimental configuration for irradiating the GNP with a laser pulse is sketched in Fig. 1a. The sample is contained in a custom-made glass cuvette with a square cross-section of width $15\,$mm and a height of $50\,$mm (Fig. 1b). The cuvette containing the liquid test sample is supported from the top and partially immersed in deionized water within a reservoir atop a medical-grade shock wave generator (Piezolith 100, Richard Wolf GmbH, Knittlingen, Germany) as also detailed in Fig. 1 of Ref.^7^. The shock wave generator consists of two spherical cap layers with piezoelectric transducers to generate transient, focused pressure waves. The focus of the spherical convergent shock wave lies within the cuvette, aligned with the lateral center of the cuvette, as illustrated in Fig. 1d. Near the focal region, the pressure wave starts with a shock front, followed by a rarefaction wave. The GNP solution in the cuvette and the liquid surrounding the shock wave generator are separated by a polyethylene film. Thus, pressure waves first propagate through the water reservoir and then couple through a thin membrane into the cuvette.

The pulse from the laser (diode-pumped Nd:YAG laser, Qantas Q2, Quantum Light Instruments) has a Gaussian intensity profile with a wavelength of 532 nm and a duration of 6 ns. It is collimated by two consecutive lenses ($\hbox {L}_1$ with $f=100\,$mm and $\hbox {L}_2$ with $f=-25\,$mm) to a beam diameter of $\sim 1.5\,$mm (full beam diameter). At time $t=0$, the laser illuminates the focal region of the rarefaction wave, as illustrated in Fig. 1c. After some delay $t=\Delta t$, the rarefaction wave reaches the laser-illuminated region (see Fig. 1d), which expands the nanobubbles present below the resolution limit of the camera into visible bubbles. Throughout our study, the delay time ($\Delta t$) is consistently maintained at 10 ms for all experiments, except for the nanobubble lifetime study presented in the final section of this manuscript. These micrometer-sized bubbles are then detected and counted from the images recorded with conventional optics. We used a high-speed camera (Photron FASTCAM AX-Mini 200 with a pixel size of $8.3\,{\upmu }\hbox {m}$) for imaging. The camera is positioned perpendicularly to the seeding laser beam. The high-speed images of the expanded nanobubbles are illuminated with a continuous white light LED through a fiber-optic light guide (SugarCUBE LED 38000-M03). All the components in the setup are triggered from a digital delay generator (BNC 525 Series, 4 channel, Berkeley Nucleonics). The experiment is automated through a lab computer.

**Figure 2:**
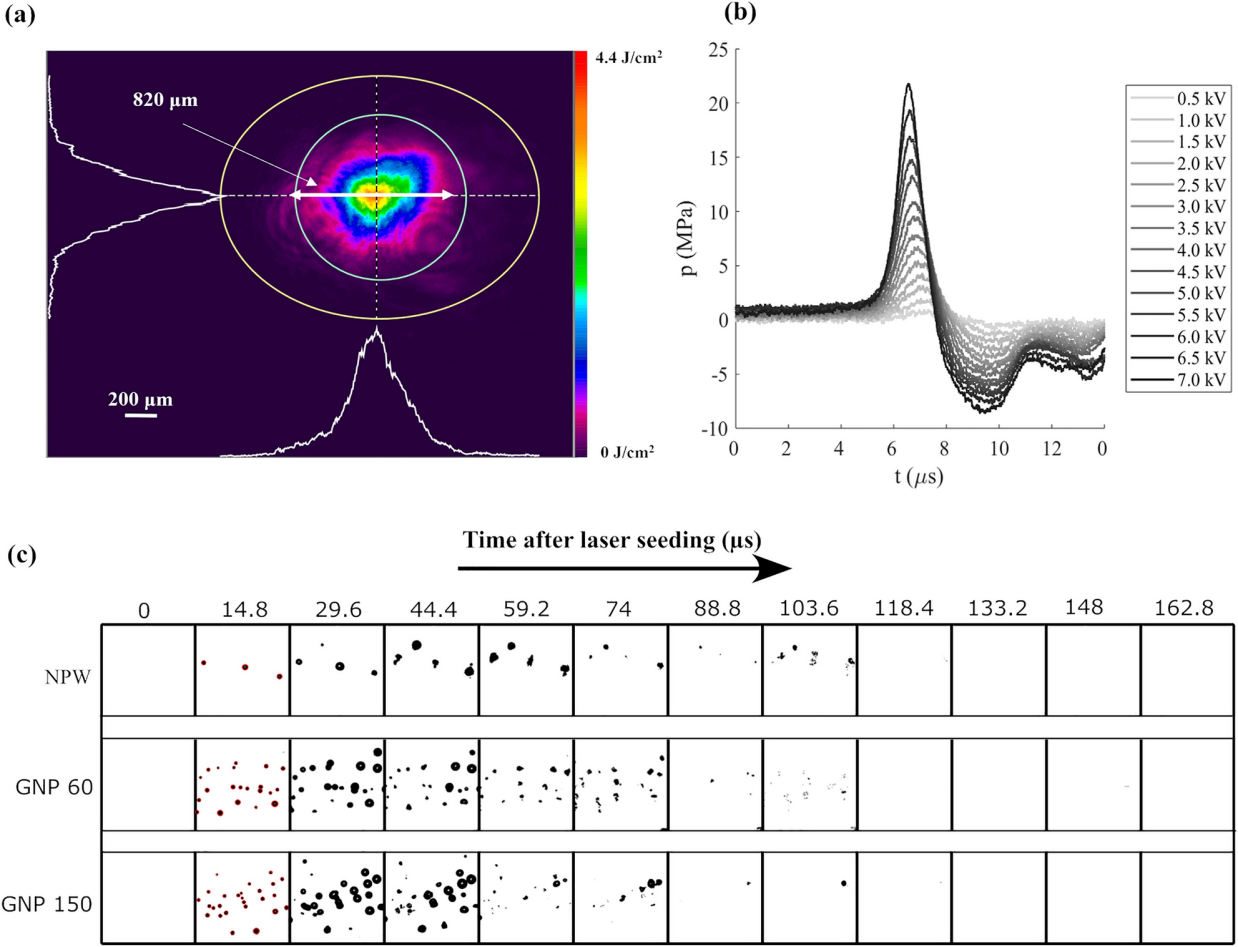
(**a**) Shape and normalized intensity distribution profile of the incident laser beam. The laser beam characterization has been done using the BeamGage Software (Version: 11455 BeamGage Standard v6.18, URL: https://www.ophiropt.com/en/g/software-download). (**b**) Pressure amplitude of the acoustic wave that drives the expansion and collapse of the bulk nanobubbles at various input voltages of the shockwave generator. (**c**) Sequence of laser-induced nanobubble generation followed by their growth and collapse dynamics under the influence of a rarefaction wave in NPW and GNP solutions containing particles of 60 and 150 nm at a concentration of $10^4$ particles/ml. The laser energy in all three cases is 19.92 mJ and the rarefaction pressure is − 5.5 MPa. The first frame following the arrival of the rarefaction wave displaying bubbles outlined in red represents the selected frame used for bubble counting, here at $14.8\,{\upmu }$s.

The laser beam’s spatial profiling and analysis were conducted utilizing the BeamGage software (11455 BeamGage Standard v6.18)^18^ and are illustrated in Fig. 2a. The dimensions of the given frame are $7.04\,\times \,5.28\, mm^{2}$ with a pixel size of $4.4\,{\upmu }$m. The overall area covered by the laser beam is depicted by the yellow ellipse, excluding noise from the outer region in the calculations. The total beam width measured here is approximately $1.5\,$mm. Within the ellipse, the green circle represents the laser beam at $1/e^2$ or 13.5 % of the maximum beam intensity, indicating a laser beam diameter of $\approx 820\,{\upmu }$m. In our study, we use this diameter to calculate the average fluence of a laser pulse. The temporal evolution of the pressure in the focal region of the shockwave (see Fig. 2b) was characterized using a fiber-optic hydrophone (HFO-690, Onda, spatial resolution $100\,{\upmu }$m and $100\,$MHz bandwidth) connected to an oscilloscope (WavePro 404HD-MS, Teledyne LeCroy, 4 GHz analog bandwidth). Detailed measurements of the bipolar pressure wave can be found in Ref.^7^ and Ref.^19^. For the settings used in this work (4 KV), we found a rarefaction pressure of − 5.5 MPa at the focus.

The acoustic focal region was determined from pressure measurements and the camera and laser were aligned accordingly. The water used in all experiments is first processed by a Milli-Q water station (i.e. deionized water filtered with a 200 nm pore-size filter) and then additionally filtered through a 50 nm pore-size filter. In this work, we refer to it as *nanopore water* (NPW). NPW was used to prepare the suspension of GNPs with diameters of 10, 60, and 150 nm (Sigma Aldrich 741957, 753653, 742058, respectively). As per the data provided by the manufacturer, all particles investigated in our study exhibit a polydispersity index of $\le 0.2$.

To evaluate the impact of individual particles on nanobubble formation and minimize interference from neighboring GNP, we selected concentrations of $3\,\times \, 10^4\,$particles/ml, $1.96\,\times \,10^4\,$particles/ml, and $3.6\,\times \,10^4\,$particles/ml for 10, 60, and 150 nm GNP, respectively. This resulted in about 110, 70, and 130 particles within the laser-illuminated volume. These concentrations were achieved by serial dilution of their respective stock solutions with initial concentrations of $6\,\times \,10^{12}$, $1.96\,\times \,10^{10}$, and $3.6\,\times \,10^9\,$particles/ml for a total sample volume of $20\,$ml.

The dynamics of the laser-induced nanobubbles in response to the rarefaction wave are depicted in Fig. 2c. The first frame of each row captures the moment of laser seeding, although the newly formed nanobubbles are too small to be detected by the camera (size below spatial resolution, $8.3\,{\upmu }$m). About $10\,{\upmu }$s after laser seeding, the rarefaction wave reaches the observation window, allowing the high-speed camera to detect the expanded bubbles in subsequent frames. The bubble dynamics in NPW and GNP solutions (with 60 and 150 nm particles) are compared under − 5.5 MPa rarefaction pressure. Despite varying nanoparticle sizes, all generated bubbles expand to about the same size because of the dominant influence of external rarefaction pressures rather than initial bubble size. Consequently, all bubbles experience similar expansion/collapse cycles under the same rarefaction pressure, regardless of their initial sizes.

## Results and discussion

### Effect of particle concentration

**Figure 3:**
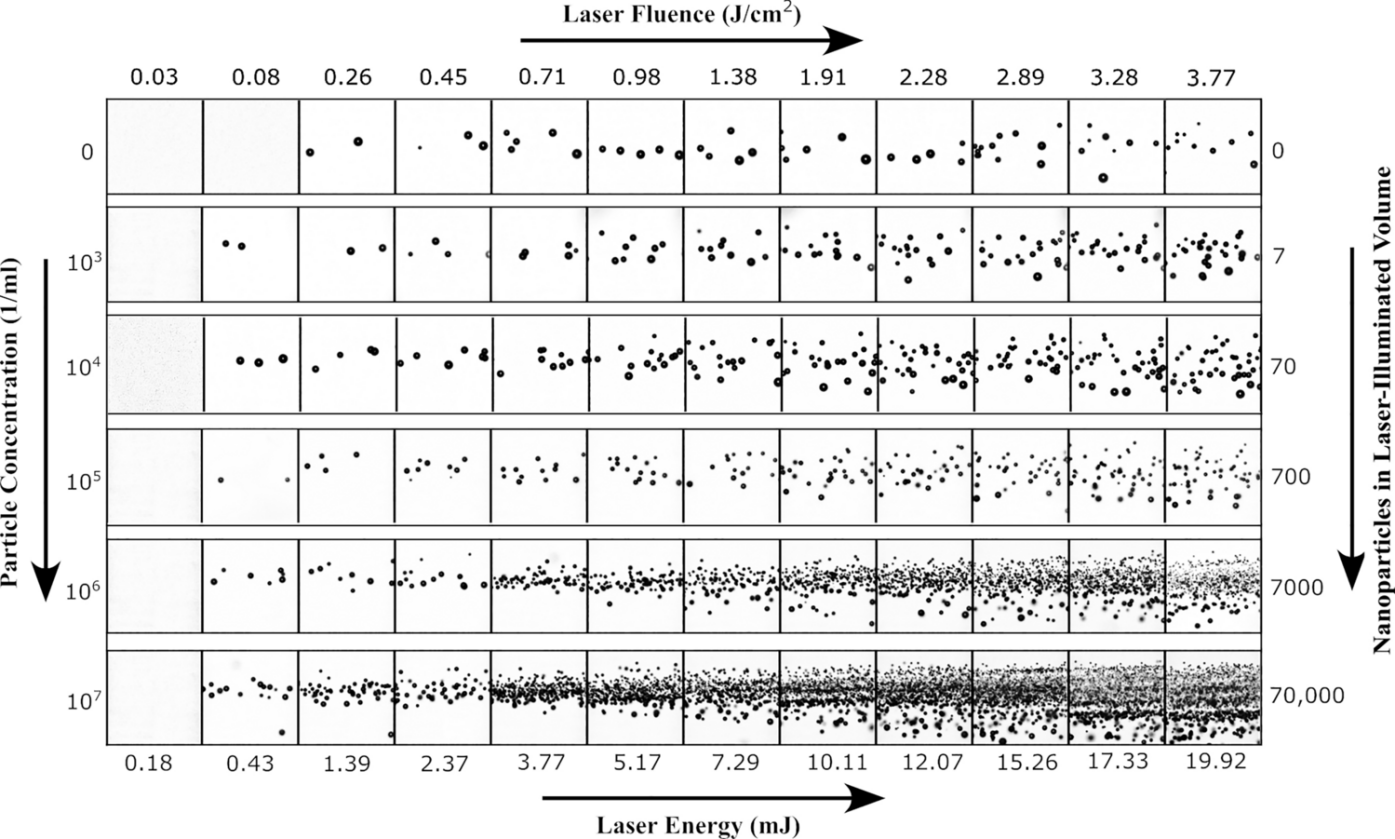
Bubble number as a function of GNP concentration and peak laser fluence or total energy. The GNP diameter is 60 nm. All frames are $2.125\,\times \,2.125\,\hbox {mm}^2$ with a pixel size of $8.3\,{\upmu }$m. The rarefaction pressure is − 5.5 MPa.

Figure 3 illustrates the relationship between the GNP concentration, laser fluence and bubble distribution for 60 nm GNPs. In this study, the concentration of GNPs ranged from $\sim 10^3-\sim 10^7\,$particles/ml, corresponding to a particle count of 7–70,000 in the total laser-illuminated volume (i.e. $\sim 3.75\, {\upmu }$L). NPW without GNPs served as the control. For all particle concentrations, the increase in bubble number with laser fluence is pronounced. The number of bubbles differed significantly from the control only at concentrations of $\sim 10^4\,$particles/ml and higher. Thus, we infer that the concentration of active particles in NPW is below this value.

For subsequent experiments, a concentration of $\sim 10^4\,$particles/ml was selected. The choice of this concentration is driven by two primary considerations: The bubble count should be low enough for reliable detection (i.e. to prevent overlapping of the expanded bubbles) and there should be a noticeable contrast in bubble count between the samples of NPW and GNP solution. As seen in Fig. 3, for low particle concentration the bubble number does not differ significantly from the bubble number in NPW, while very high concentrations lead to significant bubble overlap. Such overlaps compromise the method’s efficiency, especially when errors in counting bubbles escalate in tightly packed clusters.

### Effect of particle size

#### Fluence threshold

**Figure 4:**
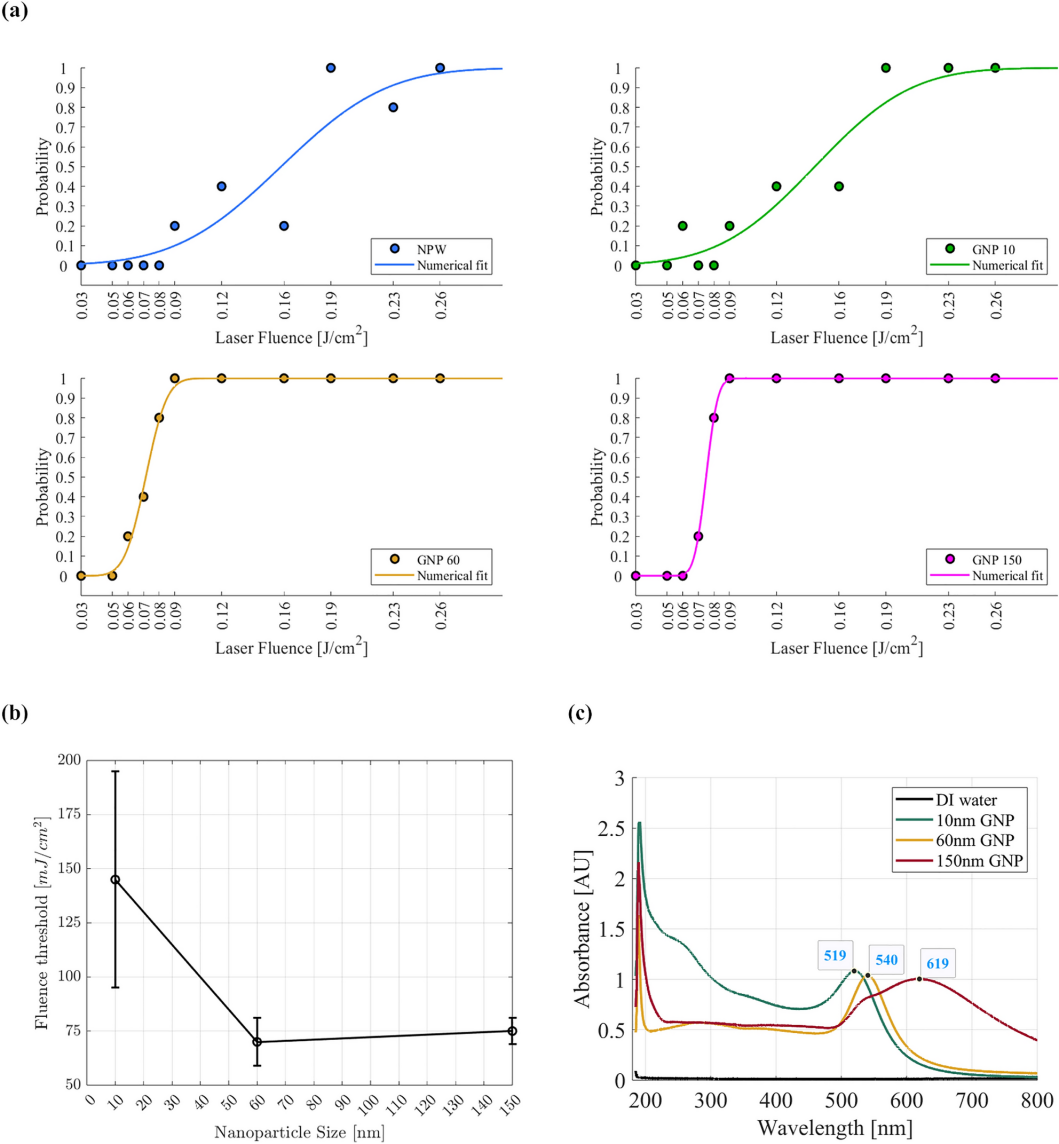
(**a**) Experimental data for the nanobubble nucleation probability as a function of laser fluence for GNP samples of 10, 60 and 150 nm diameter at a concentration of $\sim 10^4$ particles/ml. NPW is used as a control sample. The curve represents a fit to an error function. Each data point is an average of 5 measurements. (**b**) Fluence threshold as a function of GNP size. (**c**) UV-vis absorption spectra of 10, 60, and 150 nm GNPs in water.

A nanoparticle’s nucleation *fluence threshold* represents the minimum laser fluence necessary to trigger bubble formation around the nanoparticle. This threshold is influenced by various factors including the laser pulse duration, nanoparticle size (specifically its surface area-to-volume ratio), shape, material type and composition, and its surface plasmon resonance^20^. To determine the fluence threshold for GNPs of varying sizes, a statistical analysis was conducted (see Fig. 4a). In this analysis, the nucleation probability was defined as the likelihood of detecting bubble formation within the observation frame at a given laser fluence. The fluence ranged from 0.01 to $2.7\,\hbox {J}/\hbox {cm}^2$, and the nucleation probability was calculated from five repeated measurements at each fluence. An error function was then fitted to this data and the fluence at which the nucleation probability reached 50% was designated as the fluence threshold value. For NPW and 10 nm GNPs, this threshold was approximately $\approx 150\,(\pm \,50)\,\hbox {mJ}/\hbox {cm}^2$, while for 60 nm and 150 nm particles, it reduced to $70\,(\pm \,11)\,\hbox {mJ}/\hbox {cm}^2$ and $75\,(\pm \,6)\,\hbox {mJ}/\hbox {cm}^2$, respectively (see Fig. 4b). Figure 4c illustrates the surface plasmon resonance characteristics of these nanoparticles, each exhibiting an absorbance around 1 at their respective stock solution concentrations. The 10 nm and 60 nm gold nanoparticles (GNPs) resonate close to the laser wavelength used in our study (532 nm), while the 150 nm GNPs resonate at considerably longer wavelengths. Interestingly, despite the similar resonance wavelengths for the 10 nm and 60 nm GNPs, the 10 nm particles have a notably higher fluence threshold than the 60 nm particles. This indicates that additional size-dependent factors are influencing the results which will be further explored in the following sections of this paper. Furthermore, the pronounced peak around 200 nm in the far-UV spectrum (120–200 nm) results from significant energy absorption by water molecules undergoing electronic transitions within this wavelength range. It is worth mentioning that absorption is particularly sensitive to impurities, providing an effective method for estimating impurity concentrations in the sample, as highlighted by Ozaki et al.^21^.

#### Nucleation efficiency

Understanding the nucleation efficiency of the introduced GNPs requires preliminary knowledge of the terms *nucleation width* and *nucleation volume*.

**Figure 5:**
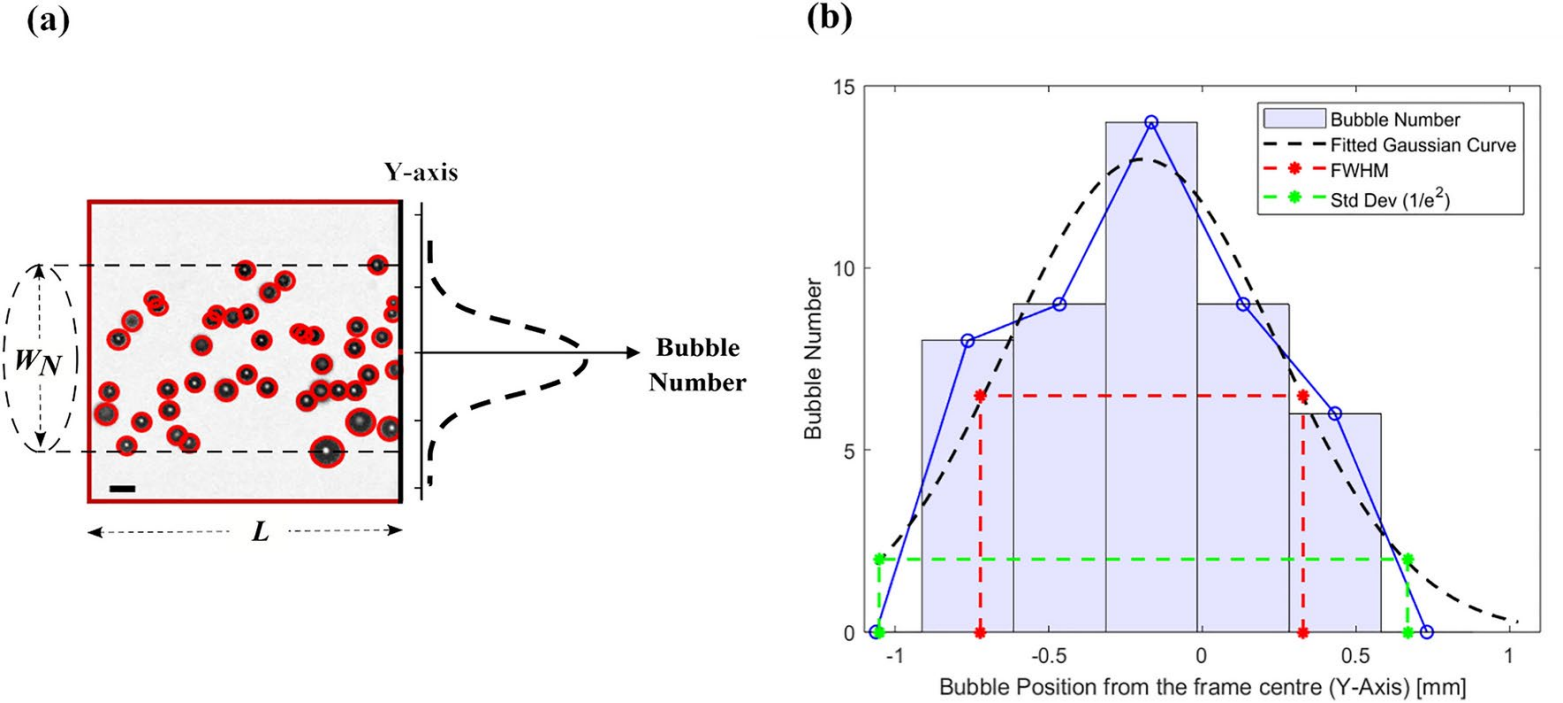
(**a**) Observation frame showing expanded bubbles within the nucleation volume ($V_N$). (**b**) Gaussian fit modeling the spatial distribution of observed bubbles along the vertical axis of the frame. The blue curve represents the bubble distribution for 150 nm GNPs at a laser fluence of $3.78\,\hbox {J}/\hbox {cm}^2$ and a rarefaction pressure of - 5.5 MPa. The bin size is $\sim 0.3\,\hbox {mm}$. The red line indicates the full width at half maximum (FWHM) of the Gaussian fit, while the green line marks the second standard deviation ($1/e^2$) of the fit, representing the effective nucleation width.

The *nucleation width* ($W_N$) refers to the effective vertical scale within the observation window where bubbles are observed for each instantaneous frame, as depicted in Fig. 5a. This width is calculated from the second standard deviation (i.e., $1/e^2$ or $13.5\%$) of the Gaussian distribution fitted to the vertical distribution of bubbles within the observation window at a given laser fluence (Fig. 5b). In this case, the chosen bin size is approximately $0.3\,$mm, with zero on the x-axis indicating the center of the frame. The frame dimensions ($L \times L$) are $2.125\times 2.125\,\hbox {mm}^2$. As the images represent the projection of bubbles distributed within a volume, the nucleation width is used as the diameter of the cylinder defining the *nucleation volume* ($V_N$):

1 $$\begin{aligned} V_N = \pi \cdot \frac{W_N^2}{4} \cdot L~. \end{aligned}$$

In other words, $V_N$ represents the cylindrical region within the overall laser-illuminated volume where the laser fluence is sufficiently high to cause bubble nucleation.

**Figure 6:**
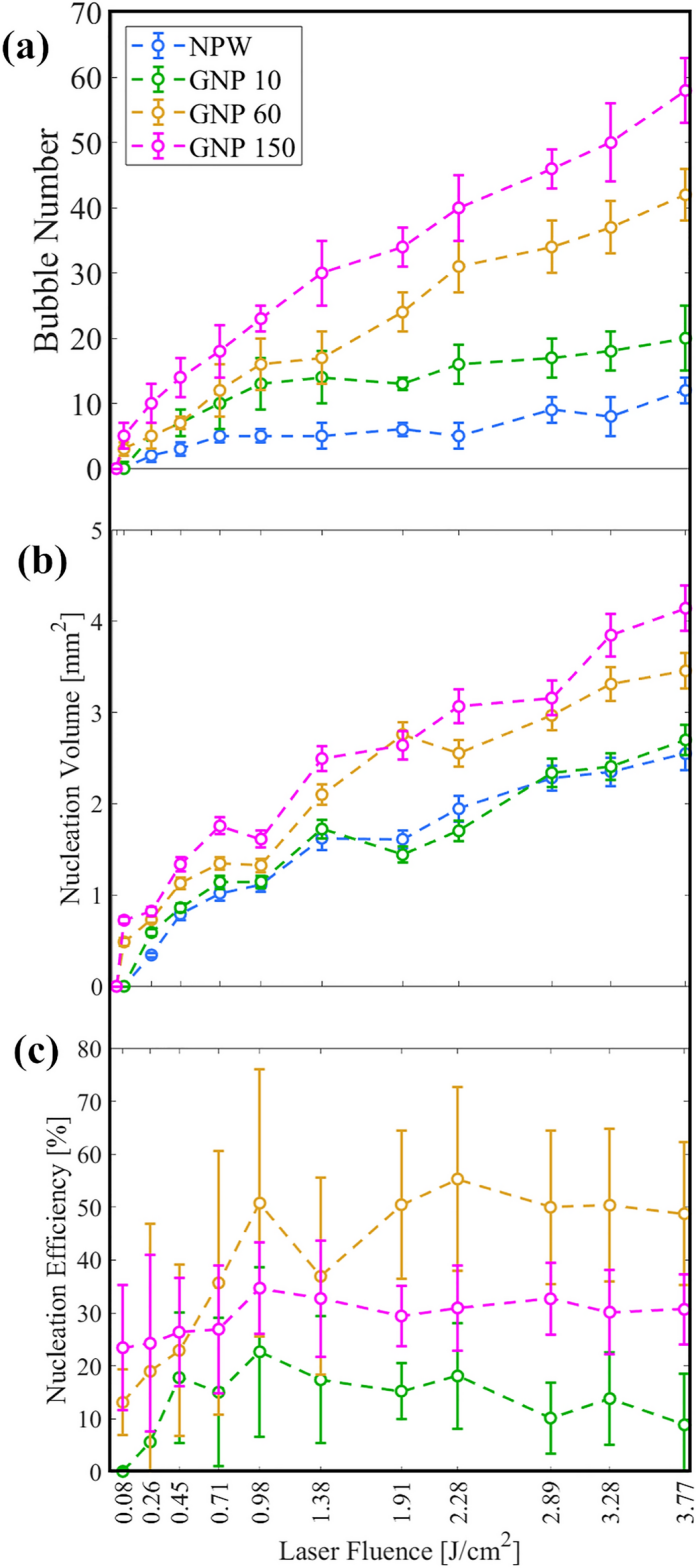
Bubble nucleation for 10, 60 and 150 nm diameter GNPs at a concentration of $\sim 10^4$ particles/ml as a function of laser fluence. NPW is used as a control. (**a**) Bubble number (**b**) Nucleation volume (**c**) Nucleation efficiency. Each data point is an average of 5 measurements, with the standard deviation indicated by an error bar. Note that the particle count in the laser-illuminated section of the observation window differs for each particle size (i.e., 110, 70, and 130 particles for 10, 60, and 150 nm, respectively).

The *nucleation efficiency* of the GNP is then calculated as the ratio of the number of bubbles observed within this volume to the number of nanoparticles it contains. Figure 6 illustrates how bubble count, nucleation volume, and nucleation efficiency vary with laser fluence for three GNP sizes: 10, 60, and 150 nm. As shown in Fig. 6a, an increase in laser fluence results in higher bubble nucleation across all samples, irrespective of particle size and concentration. However, variations in bubble counts based on particle sizes are influenced by two key factors: the particle count in the laser-illuminated volume and their specific fluence threshold for nucleation. Within the laser-illuminated volume, there are 110, 70, and 130 particles of 10, 60, and 150 nm sizes, respectively. The 150 nm GNPs, which have a slightly higher fluence threshold, nonetheless display a greater bubble count (Fig. 6a) and an expanded nucleation volume (Fig. 6b) in comparison to the 60 nm GNPs, likely due to their marginally higher concentration. Conversely, 10 nm GNPs, despite their higher particle count, exhibit a nucleation volume comparable to that of NPW, suggesting their small influence on nanobubble formation. As depicted in Fig. 6c, the 60 nm GNPs achieve the highest nucleation efficiency at approximately 45%, while the 10 nm and 150 nm particles yield efficiencies of 15% and 30%, respectively. Nucleation efficiencies for all GNPs remain fairly constant across fluences because with increasing laser fluence, more regions farther from the laser center surpass the threshold for bubble formation, thereby expanding the nucleation volume. This allows more nanoparticles within the enlarged region to receive sufficient energy needed for participating in nanobubble formation, increasing the overall bubble count. However, below the fluence of $0.45\,\hbox {J}/\hbox {cm}^2$, 60 nm GNPs display a relatively lower nucleation efficiency than 150 nm GNPs (Fig. 6c). This discrepancy is likely due to the smaller number of 60 nm GNPs within the nucleation volume, which increases measurement uncertainty.

The maximum laser intensity in our experiments, achieved at a laser fluence of $3.77\,\hbox {J}/\hbox {cm}^2$ for a 532 nm laser pulse with a duration of 6 ns, reaches approximately $0.6\,\hbox {GW}/\hbox {cm}^2$. This intensity is significantly lower, by at least two orders of magnitude than the reported laser-induced dielectric breakdown threshold in water, $I_{thresh} \approx 30\,\hbox {GW}/\hbox {cm}^2$. Unlike all previous studies on this topic that primarily utilized focused laser beams to heat nanoparticles for plasmonic nanobubble generation, our work is the first to employ a collimated laser beam at intensities much below the threshold for multiphoton ionization. This suggests that the nanobubble generation is not a result of optical breakdown but rather the linear absorption of laser energy by the GNPs.

### Temperature modeling

Bubble nucleation is governed by three key processes in laser energy transfer from plasmonic nanoparticles: plasmonic laser energy absorption (linked to the absorption cross-section), conversion of the absorbed energy to heat (thereby raising the nanoparticle’s temperature, dependent on the particle’s heat capacity), and heat dissipation into the surrounding medium (influenced by the particle volume, the temperature gradient at the particle-liquid interface, and the thermal conductivity of the liquid)^13^. In the context of thermoplasmonic nucleation, the success of vapor bubble formation depends on the nanoparticle’s thermal exchange efficiency, defined as the ratio of the energy dissipated across the particle-liquid interface to the total energy absorbed by the particle^22^. This efficiency is closely tied to the particle’s fluence threshold.

**Figure 7:**
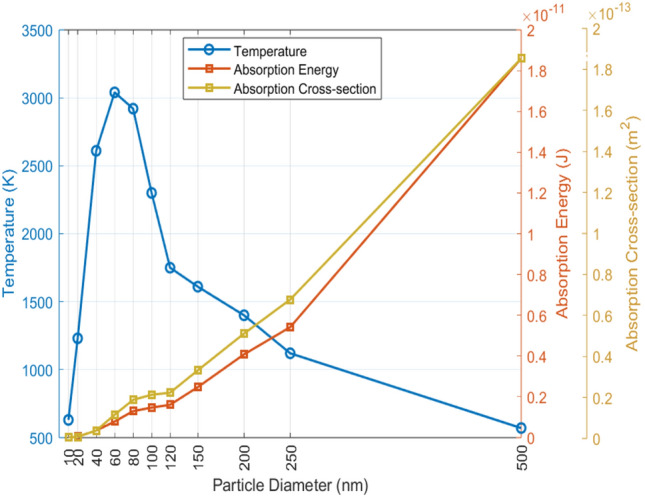
Calculated temperatures and absorption energies of GNPs at their respective thresholds for bubble nucleation as a function of particle diameter.

The absorbed heat is transferred from the nanoparticle to the surrounding liquid, which can trigger the vapor bubble formation for a pulsed laser at a sufficiently high fluence. This vapor bubble surrounding the particle alters the refractive index, increasing the scattering of incident light and subsequently reducing the energy absorption on the particle’s surface^20^. This diminished absorption of incident light may result in significantly lower nanoparticle temperatures, although still sufficient for ongoing vaporization of the surrounding medium, depending on the duration of the laser pulse. Metwally et al.^23^ suggest that the nonlinear relationship between the particle’s absorption cross-section and its volume, along with the thermal exchange resistance (Kapitza resistance, $1/g$) at the particle-liquid interface, may impact nanoparticle temperatures and consequently, fluence thresholds. However, they rule out a significant effect of the Kapitza resistance for particles smaller than 200 nm diameter in the nanosecond pulse regime. They argue that in this regime, heating times ($\tau _p \approx 1-10\,$ns, specifically 6 ns in our case) for small nanoparticles are much longer than the heat dissipation times with interfacial resistance ($\tau _g$), which span a few picoseconds to few nanoseconds for particles between 10 and 200 nm. As a result, small nanoparticles dissipate heat rapidly, creating a quasi-static regime where Kapitza resistance has minimal impact on the surrounding medium. For larger particles, however, $\tau _g$ becomes comparable to $\tau _p$, slowing heat release and making interfacial resistance more influential on the surrounding medium. Using the programs provided by Metwally et. al. in Ref.^23^, we computed the anticipated absorption energies required for nanobubble nucleation and the corresponding temperatures for GNPs of diameters ranging from 10 nm to 500 nm (see Fig. 7). The program relies on Mie theory for the calculation of the absorption cross-section and employs the Runge-Kutta-4 method to discretize the temporal and spatial evolution of temperature in the particle and water. Here, we used the parameters provided in Ref.^23^ with a laser pulse duration of 6 ns.

Figure 7 illustrates that both the absorption coefficient and the absorbed energy increase with particle size due to their direct relationship with the particle’s surface area. However, temperatures initially rise as the particle size increases from 10 to 60 nm and then decrease with further increases. This shift in trend is associated with the increase of the particle volume with its size and the decrease in the surface area-to-volume ratio. Since the particle’s temperature depends on its surface area-to-volume ratio, more energy is required to raise the particle temperature with a greater heat capacity. Consequently, maximum temperatures are attained at a particle diameter of 60 nm. Based on these computations, the absorption energies of GNPs with sizes of 10, 60, and 150 nm at their respective laser fluence thresholds of $145\,(\pm \,50)\,\hbox {mJ}/\hbox {cm}^2$, $70\,(\pm \,11)\,\hbox {mJ}/\hbox {cm}^2$, and $75\,(\pm \,6)\,\hbox {mJ}/\hbox {cm}^2$ are determined to be $6.67 \cdot 10^{-15}\,\hbox {J}$, $7.84 \cdot 10^{-13}\,\hbox {J}$, and $2.47 \cdot 10^{-12}\,\hbox {J}$, respectively, while their corresponding maximum temperatures are about $610\,(\pm \,210)\,\hbox {K}$, $3040\,(\pm \,470)\,\hbox {K}$, and $1610\,(\pm \,130)\,\hbox {K}$. A key point to note here is that the GNPs are uniformly dispersed throughout the liquid sample and are undergoing Brownian motion, making it unlikely for the same particles to repeatedly intersect the laser path across multiple trials. While high nanoparticle temperatures (as depicted in Fig. 7 can potentially reshape nanoparticles into a spherical form after repeated laser exposure, as observed by^11^, such transformations do not significantly alter the particle diameter. Furthermore, our analysis does not depend on particle shape, ensuring that any structural changes due to melting will not influence bubble formation. This consistency supports the reliability of our experiment, allowing us to rule out nanoparticle melting and its potential effects as factors influencing our results.

The above calculations show that as the nanoparticle size decreases, its reduced heat capacity and increased surface-to-volume ratio result in lower energy absorption and more effective heat dissipation into the surrounding medium, leading to faster cooling of the nanoparticle. Therefore, a higher fluence is needed to counterbalance the quicker cooling and reach the threshold for bubble formation. Conversely, larger nanoparticles have an increased heat capacity, and thus a reduced optical absorption per volume. Consequently, the fluence threshold also increases with the particle size. The combination of these opposing trends leads to the occurrence of a minimum in the fluence threshold concerning nanoparticle size. In our case, the minimum was observed for the 60 nm particles (see Fig. 4b).

The findings were consistent with studies performed under similar experimental conditions^15,22,24,25^, but showed at least a two-order of magnitude difference from others^26,27^ under comparable conditions. Some of these studies^15,26^ used high-energy-density focused laser beams to induce dielectric breakdown in water, resulting in nanobubble formation. Consequently, threshold determination relied on either macroscopic bubble observations or scattering techniques for nanobubble detection. However, these methods introduce uncertainties in detection, leading to higher measured fluence thresholds. On the contrary, our method employs a collimated beam for nanobubble generation, offering greater precision by detecting early liquid phase changes around the particles. This precision allows us to achieve bubble generation at lower energies, explaining the observed lower fluence threshold.

### Bubble lifetime and size distribution

**Figure 8:**
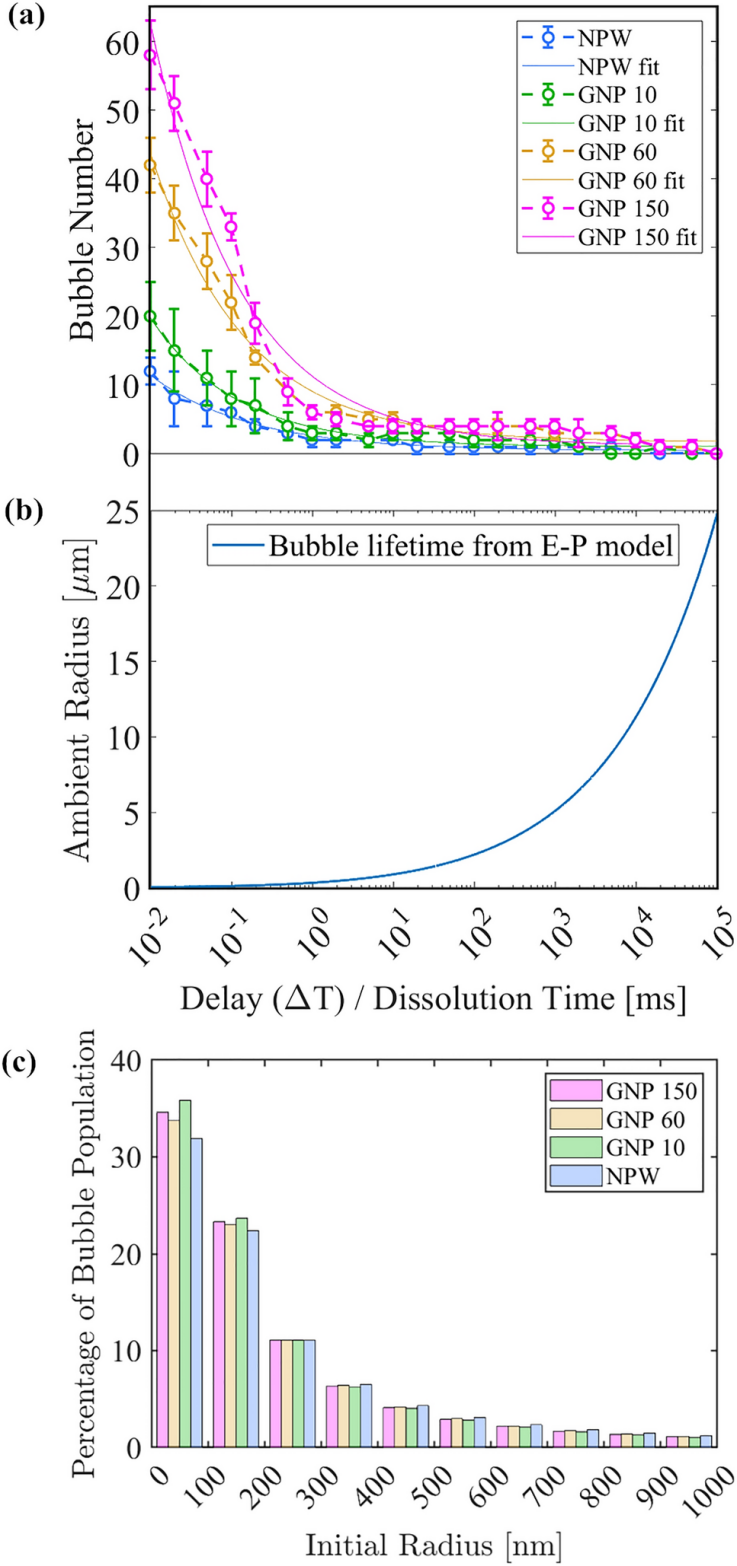
(**a**) Mean number of bubbles remaining in the liquid as a function of time delay between laser seeding and the arrival of the rarefaction wave ($\Delta$t) with the function ($ae^{-b{\Delta }t}+c$) fitted to the measured data. (**b**) Bubble lifetime as a function of its initial ambient radius computed for water at room temperature using the Epstein-Plesset equation. (**c**) Bubble number as a function of the initial ambient nanobubble radius. The laser energy is $(19.92 \pm 0.02)$ mJ and the rarefaction pressure is $(-5.5 \pm 0.2)$ MPa. Each value represents an average of 5 measurements.

The lifetime of a nanobubble is defined as the time between its formation and its complete dissolution. It depends on factors such as its ambient size (i.e. its equilibrium size at rest), the surrounding gas concentration, surface properties, and environmental conditions like temperature and pressure. According to the Epstein-Plesset theory^28^, since surface tension increases a bubble’s interior gas pressure as compared to its environment (by an increment known as the Laplace pressure), small bubbles tend to dissolve due to gas diffusion. Therefore after their inception, the number of nanobubbles present in our sample is expected to decrease over time. In our study, we leveraged this principle by introducing a time delay between the laser-induced nanobubble generation and their visualization upon expansion by a rarefaction wave. This approach allowed us to conclude that bubbles observed after a specific delay time ($\Delta$t) have lifetimes longer than the time delay, enabling an estimate of their initial sizes. The results, presented in Fig. 8a, reveal a rapid decline in the bubble population within $1\,\hbox {ms}$ after laser seeding. We fitted the data numerically using the function $ae^{-b\Delta t}+c$ to model the observed decline. To characterize the dissolution dynamics of the generated bubbles, we utilized the Epstein-Plesset equation given by Plesset and Sadhal^28^.

Figure 8b shows the calculated lifetimes for bubbles in water with initial radii ranging from 20 nm to 25 µm. For comparison with Fig. 8a, we have inverted the plot to display initial radii corresponding to their respective calculated lifetimes. Bubbles visible at a given delay $\Delta t$ thus have lifetimes beyond $\Delta t$, allowing us to deduce that their initial radii exceed the radius associated with a lifetime of $\Delta t$ in Fig. 8b. By establishing a correlation between the fitted bubble count as a function of the delay time ($\Delta$t) and the dissolution time calculated from the Epstein-Plesset equation, we derived the size distribution of the bubbles generated (Fig. 8c). The obtained bubble size distribution confirms the nanometric size of the bubbles. Notably, about 70% of the bubbles produced by the laser had an initial ambient radius below 300 nm, while 90% of the bubbles were smaller than 1000 nm in radius.

Despite the 60 nm particles exhibiting the highest nucleation efficiency (Fig. 6), there was no notable impact of particle size on the bubble lifetime (Fig. 8). This lack of influence is attributed to the size of the vapor bubble around nanometric-sized particles being primarily dependent on the duration of the incident laser pulse^20^, which remained constant at 6 ns in our experiments. One possible explanation for the formation of gaseous nanobubbles is the accumulation of gas through diffusion, enabling the initial vapor bubble to expand. Previous studies^29,30^ have investigated the impact of gas concentration in the surrounding liquid on the expansion of plasmonic nanocavities into micron-sized bubbles generated using continuous-wave (CW) laser sources with prolonged exposure times. However, this phenomenon has not been extensively studied for pulsed lasers, as the short timescales of mass transfer processes during the laser excitation, bubble nucleation, expansion, and subsequent collapse introduce more complex dynamics. Additionally, as the observed dissolution times align with the Epstein-Plesset model’s predictions for bubbles smaller than $\mathcal {O}(1 \, {\mu}m)$, the potential influence of stabilization mechanisms such as surface charges on the bubbles was considered negligible.

## Conclusion

In this study, we have advanced a novel technique introduced by our team for on-demand nanobubble generation using a collimated laser beam. In our previous studies^5,7^, we suggested that nano-sized impurities in the water might induce the formation of nanometric bubbles through a phase transition triggered by the linear absorption of laser light. However, the precise origin of the nanobubbles was unclear. In this study, we focused on the effect of deliberately introduced gold nanoparticles (GNPs) as impurities to better understand their role in nanobubble formation. Additionally, we explored how varying particle sizes and concentrations can optimize nanobubble generation at laser intensities well below the multiphoton ionization threshold. The relationship between GNP concentration and nanobubble nucleation showed a sub-linear pattern, with a significant deviation in bubble count from the control observed only at concentrations of $\sim 10^4\,$particles/ml and above. While the exact reason for this abrupt change is not fully elucidated, it merits further exploration in future research. Additionally, the 60 nm particles exhibited the lowest fluence threshold of $70\,(\pm \,11)\,\hbox {mJ}/\hbox {cm}^2$ and the highest nucleation efficiency of $\approx$45 %. We explain the increase in fluence threshold for small GNPs (10 nm) with rapid energy release, while for large GNPs (150 nm) it is attributed to the non-linear relationship between the absorption cross-section and the GNP volume, though there was only a small difference in the fluence threshold between 60 nm particles and the 150 nm. Despite the enhanced nucleation efficiency of the 60 nm particles, the lifetime of the bubbles does not show a strong dependence on the particle size. Although we confirm that bubble nucleation from plasmonic heating is essential for nanobubble formation, we have not answered the question of how the nanobubbles become gaseous. We speculate that gas diffusion from the liquid surrounding the nucleated cavities is a relevant factor not only in the nanobubble formation process but also in their dissolution dynamics. Future experiments using water with different dissolved gases and varying gas concentrations could verify this hypothesis. However, our experimental setup does not currently allow for precise control of gas concentrations in the liquid, which presents challenges for data interpretation. To address these issues, we are developing appropriate models that will be detailed in a forthcoming manuscript. The method used in this study not only serves as a reliable tool for nanobubble lifetime characterization after the nucleation event but also provides a framework for understanding the efficiency of nanoparticles in generating nanobubbles. This understanding is essential for optimizing applications that rely on controlled nanobubble formation, such as targeted drug delivery and diagnostic imaging.

## Data Availability

The data supporting the findings of this study are available from the corresponding author on a reasonable request.
